# Vitamin D and Anti-Müllerian Hormone Levels in Infertility Treatment: The Change-Point Problem

**DOI:** 10.3390/nu11051053

**Published:** 2019-05-10

**Authors:** Anna Bednarska-Czerwińska, Katarzyna Olszak-Wąsik, Anita Olejek, Michał Czerwiński, Andrzej Tukiendorf

**Affiliations:** 1 GynCentrum Clinic, Żelazna 1, 40-851 Katowice, Poland; annabednarska@post.pl (A.B.-C.); czerwinski.m92@gmail.com (M.C.); 2 Department of Gynecology, Obstetrics and Oncological Gynecology, Silesian Medical University, Batorego 15, 41-902 Bytom, Poland; anitaolejek@wp.pl; 3 Department of Public Health, Wrocław Medical University, Bartla 5, 51-618 Wrocław, Poland; andrzej.tukiendorf@gmail.com

**Keywords:** vitamin D, anti-Müllerian hormone, follicular fluid, segmented regression

## Abstract

Background: Anti-Müllerian hormone (AMH) is considered to be one of the most significant indicators of women’s fertility. Many studies have shown that vitamin D may modify human reproductive functions; however, the results are conflicting. The composition of follicular fluid (FF) creates the biochemical environment of the oocyte and affects its quality, which later determines the embryo quality. In this study, we aimed to revise with advanced statistical techniques the relationship between AMH and vitamin D in FF. Methods: The study was designed as a prospective single-center study in infertile patients with AMH ≥ 0.7 ng/mL who underwent controlled ovarian hyperstimulation for in vitro fertilization. AMH and vitamin D levels in FF were measured. Next, the standard and advanced statistical (including segmented regression) techniques were applied. Results: We observed a negative linear correlation between levels of AMH in serum and FF and total vitamin D concentrations up to approximately 30 ng/mL; with a statistically significant relationship in FF. Beyond that concentration, the trend was positive but statistically insignificant. Conclusions: As an existing “change-point problem” was noticed, we suggest segmentation in the relationship between vitamin D and AMH during infertility treatment.

## 1. Introduction

It is widely known that the anti-Müllerian hormone (AMH) in the serum of women of reproductive age is secreted by the granulosa cells of ovarian follicles and appears to regulate early follicle development. Its level varies slightly with the menstrual cycle, reaching the peak value during the late follicular phase [1]. However, the serum AMH level varies greatly among similar individuals for unknown reasons [2]. Notably, when estradiol and follicle-stimulating hormone (FSH) levels follow the expected patterns during gestation, no significant modifications are found in AMH levels during pregnancy or in the early puerperium; thus, FSH does not seem to play a direct role in AMH synthesis and secretion [1]. Moreover, the excessive production of AMH by growing follicles is now considered to be an important feature of polycystic ovary syndrome (PCOS). The lack of an international standard for the serum AMH assay, mainly because of technical issues, makes it difficult to define consensual thresholds and thus impairs the widespread use of this new ovarian marker in the diagnosis of PCOS [3]. It is hoped that the serum AMH assay will soon be improved for predicting menopause. Based on standard statistical methods, the relationship between ovarian reserve tests and time to menopause were recently defined [4]. A reduction in AMH with age in women has also been confirmed by some investigators [5].

The skin, liver, and kidneys are the main organs involved in the production of vitamin D and its metabolites. First, hydroxylation of vitamin D synthesized in the skin takes place in the liver. Next, hydroxylation in the kidneys turns 25-hydroxyvitamin D (25(OH)D) into the active 1,25-dihydroxyvitamin D3 form (1,25(OH)_2_D_3_). In addition, other tissues (e.g., placenta) are able to produce 1,25(OH)_2_D_3_ [6]. Most 25(OH)D is bound to the vitamin D binding protein (DBP) or to serum albumin. Only around 1% of 25(OH)D acts as a free biologically active hormone [7]. For routine diagnostic testing, total 25(OH)D is measured.

It is known that vitamin D deficiency may be a factor participating in the development of gestational diabetes mellitus, as vitamin D regulates insulin production and insulin response in tissues. It is believed that maternal supply of vitamin D affects early fetal development and fetal-placental immune response which, if inappropriate, entails the risk of preeclampsia [6]. Vitamin D status in pregnancy also impacts the offspring’s long-term health outcomes (bone development, birth and postnatal weight, tendency for autoimmune diseases, and neuropsychiatric outcomes). The biologically active 1,25(OH)_2_D_3_ metabolite increases with the gestational age; however, this rise may result from the availability of 25(OH)D [6].

It is also hypothesized that vitamin D has a direct effect on AMH production, and, thus, patients with higher concentrations of vitamin D are able to maintain their ovarian reserve for longer [8]. Studies either confirm that vitamin D may be a positive regulator of AMH production [2], or report that its levels are unrelated to ovarian reserve or ovarian response after ovarian stimulation [9]. Significant seasonal variations in serum vitamin D were observed between summer and winter; however, serum AMH levels remained unaffected by season [10]. A large review examined the role of vitamin D in ovarian physiology and its implications for reproduction [11]. Follicular fluid (FF) serves as the biochemical microenvironment of the oocyte before ovulation. Among its constituents, vitamin D was studied in terms of possible influence on female fertility [12]. To date, however, no negative relationship between vitamin D concentration in the serum and FF has been found in the literature.

Given the above facts, the aim of this clinical study was to update current knowledge regarding the relationship between vitamin D levels and levels of AMH in serum and FF. Our purpose was to identify changes of vitamin D and AMH concentrations and then to estimate the possible location of changes (change-points). Standard and advanced statistical techniques were applied in the statistical analysis to investigate “the change-point problem”.

## 2. Material and Methods

### 2.1. Study Design and Participants

The present study was designed as a prospective single-center cohort study comprising 53 women with infertility (Caucasian ethnicity, with secondary or university levels of education, aged 34.7 ± 4.1 years, with a mean body mass index (BMI) of 22.2 ± 2.7 kg/m^2^) who sought medical attention at GynCentrum, Katowice, Poland, in 2017. All were diagnosed with tubal factor infertility. Based on this diagnosis, couples were qualified for in vitro fertilization (IVF). Only patients in good general health condition were asked to participate in the study. Patients with a medical history of hypertension, diabetes, renal dysfunction, hyperinsulinism, PCOS, and endometriosis were not enrolled in the study.

### 2.2. Ethical Consideration

All the patients signed an informed consent form approved by the Silesian Medical University Ethics Committee (ref. no. KB1/63/16). All methods were performed in accordance with the guidelines of the European Society of Human Reproduction and Embryology, the American Society for Reproductive Medicine, and the Polish Society of Reproductive Medicine and Embryology.

### 2.3. Data Collection

Before undergoing infertility treatment, all the recruited patients provided a blood sample on the day of their first consultation, irrespective of the day of the menstrual cycle (for the AMH test). Between the second and third day of the patient’s menstrual cycle, prior to ovarian hyperstimulation, additional hormone tests were performed (FSH-follicle stimulating hormone, LH- luteinizing hormone and estradiol). All patients with AMH ≥ 0.7 ng/mL were scheduled to undergo controlled ovarian hyperstimulation (COH) using the antagonist protocol, intracytoplasmic sperm injection, and a single embryo transfer. The starting dose of COH was based on age, AMH, FSH, BMI (body mass index), and experience from previous cycles. Gonadotropin (Gonal F, Merck Serono, UK, and Menopur, Ferring, Germany) doses were further adjusted according to ultrasound findings and estradiol measurements during stimulation monitoring. When at least three follicles reached a diameter of 18 mm, recombinant human chorionic gonadotropin (r-hCG) (Ovitrelle, Serono, Switzerland) was administered, and 35–36 h later, oocyte retrieval was performed under light sedation. FF from the first mature follicle aspirated was collected before any flushing, for oocyte recovery. The FF was then centrifuged at 3000 cycles/min and stored at −60 °C until assayed.

All laboratory measurements were performed using an electrochemiluminescent (ECLIA) immunoanalyzer (Cobas e411, Roche Diagnostics, Mannheim, Germany). Vitamin D and anti-Müllerian hormone levels in the FF were measured according to the quantitation limits of the assay at 3.0 and 0.1 ng/mL. Serum AMH samples were obtained at the first consultation, irrespective of the day of the menstrual cycle. Blood was drawn in plain serum tubes, centrifugation was performed within 1 h, and then the serum was separated. The results were measured using the immunoanalyzer manufacturer’s instructions.

### 2.4. Statistical Analysis

The initial design of the study was confirmed using a calculation to determine the sample size needed to ensure that a correlation coefficient between vitamin D levels and AMH will differ from zero. In the next step, a Student’s *t*-test was applied to show seasonal differences between the first (winter + spring) and the second (summer + autumn) semesters, and Pearson’s correlation was used to illustrate linearity between AMH in serum and FF and vitamin D levels. Finally, to identify changes in levels of AMH in serum and FF at unknown vitamin D concentrations, and to estimate the location of changes, change-point regression was used. Generally, change-point regression (also called segmented regression) is a regression in which the expected value of the dependent variable or response is assumed to have a different functional form in several neighborhoods of the explanatory variable space. The change-point is estimated in so-called broken-line regression models, where the regression function is assumed to be continuous at the point of change. Numerous methodological approaches have been implemented to examine change-point models; typically, these involve maximum-likelihood estimation or Bayesian estimation [13].

## 3. Results

### 3.1. Sample Size Calculation

This statistical procedure showed that the collected sample size of 53 patients had a power, or 1 - β (probability of failing to reject the null hypothesis under the alternative hypothesis = type II error rate), of more than 90% to detect a correlation between vitamin D levels and AMH levels with *r* = 0.1 (the expected correlation coefficient) and a significance level α (threshold probability for rejecting the null hypothesis = type I error rate) of 0.05 using a two-sided *Z*-test [14].

### 3.2. Student’s t-Test

First, seasonal differences between the first (winter + spring) and the second (summer + autumn) semesters were evaluated using a standard Student’s *t*-test. The results are displayed in Table 1. 

**Figure 1:**
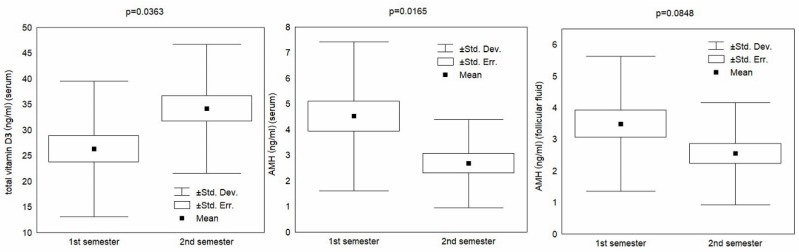
Total vitamin D and anti-Müllerian hormone (AMH) by semester.

A statistically significant difference (*p* < 0.05) in total vitamin D and AMH levels in serum between the semesters was established (Table 1). Regarding the AMH levels in FF, the difference bordered on statistical significance (*p* < 0.1). The results are displayed graphically in Figure 1.

Then, following the report issued by the Endocrine Society (ES) [15] regarding norms for the minimum blood level of vitamin D (suggested vitamin D norm of 30 ng/mL) for the selected variables, the same standard Student’s *t*-test was performed to identify significant differences between the subgroups of women. The results are reported in Table 2.

**Figure 2:**
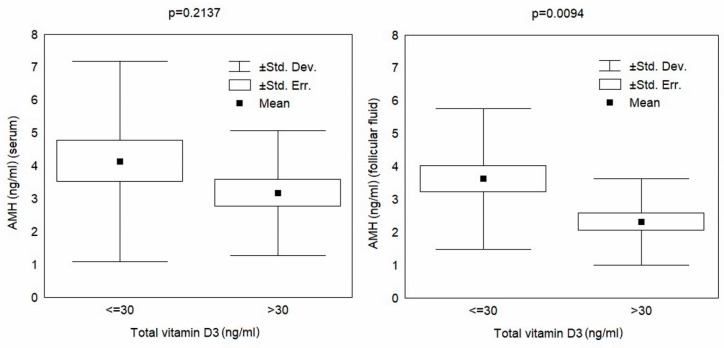
AMH (anti-Müllerian hormone) in serum and in follicular fluid relative to the vitamin D norm (30 ng/mL).

The Student’s *t*-test results shown in Table 2 indicate an insignificant difference in the AMH concentration in serum in reference to the ES norm, but a statistically significant decrease in the AMH level in the FF among patients with higher levels of vitamin D. The results are plotted in Figure 2.

### 3.3. Pearson’s Linear Correlations

The results of Pearson’s linear correlations for AMH, in serum and FF, and vitamin D levels are reported in Table 3.

**Figure 3:**
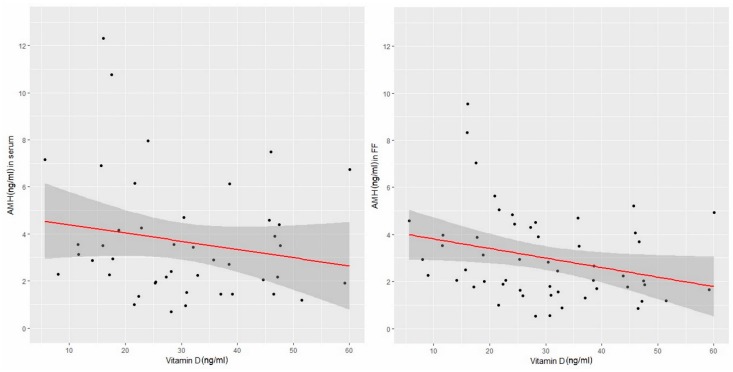
AMH versus vitamin D in serum and in follicular fluid (linear correlations). (AMH- anti-Müllerian hormone, FF- follicular fluid).

The data presented in Table 3 indicate both the negative linear correlations between AMH in serum and FF and the total vitamin D concentration. However, the relationship between AMH levels in FF and vitamin D concentration was statistically significant. The results are presented graphically in Figure 3.

Figure 3 shows that some apparent change-points for the AMH concentration, in both serum and FF, occur around a vitamin D concentration of 30 ng/mL. A possible segmentation of this relationship will be checked using the appropriate statistical tool (i.e., segmented regression).

### 3.4. Change-Point Problem

In this study, the “segmented” R package [16] was used to estimate trends in the change-points and slopes of AMH (in serum and in FF) in relation to the total vitamin D concentration (Table 4).

**Figure 4:**
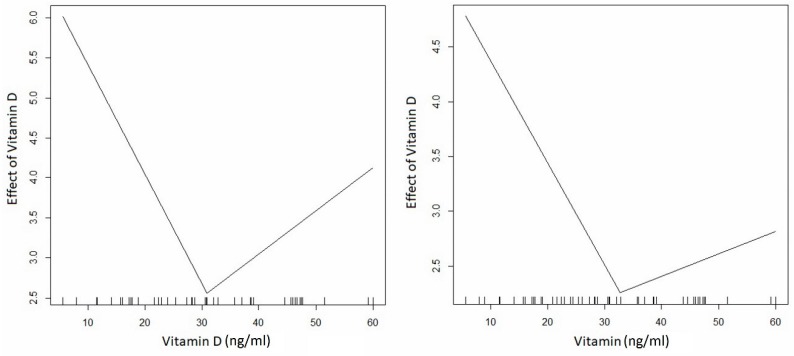
Effects of vitamin D on AMH in serum and follicular fluid.

The results reported in Table 4 were interpreted as follows: negative effects of vitamin D on AMH in serum and FF were observed at concentrations up to 31 and 33 ng/mL, respectively. In the serum, the effect was borderline significant, whereas in the FF, the impact was statistically significant. The larger the estimated change-point vitamin D concentrations, the more positively they influenced the analyzed AMH trends; however, the effects were statistically insignificant (see Table 4, for details). The effects of vitamin D concentrations can be observed in Figure 4.

## 4. Discussion

There is an increasing awareness that vitamin D plays an important role in reproduction, and several clinical studies suggest a correlation between adequate vitamin D levels and successful fertility treatments in women with infertility [11] (mostly in the context of in vitro fertilization) [17,18]. Because seasonal fluctuations in serum vitamin D levels are related to ultraviolet light exposure [19] and because the “sunshine vitamin” is related to seasonal fluctuations in ovulation [20], it is reasonable to hypothesize that vitamin D may be capable of influencing ovarian function and AMH production [10]. Some investigators have even observed a positive linear relationship between vitamin D and AMH [2]. It is also believed that acute supplementation with high-dose vitamin D rapidly increases serum AMH in young women [21]. However, it seems that dietary intake contributes to vitamin D blood levels to a lesser extent.

AMH inhibits the primordial to primary follicle transition. This repressive effect on granulose cell differentiation is mediated by highly specific type II receptors (AMHR-II); thus, higher AMH is responsible for suppressing follicles maturation. The role of vitamin D is to inhibit AMHR- II [22]. A study by Malloy et al. [23] also showed that the AMH promoter contains a functional vitamin D response element (VDRE) and its expression is regulated by 1,25(OH)_2_D_3_. The physiological relevance of AMH is unclear [24]. In a study of rhesus macaque follicles, AMH concentrations were higher in the lower vitamin D media (25 pg/mL) during 5 weeks of culture than in the higher vitamin D media (100 pg/mL). Given that the effect was reversed at 4 weeks, the action of vitamin D on the primate follicle appears to be indirect [25]. A critical appraisal of the role of the “sunshine vitamin” can also be found in a paper by Laganà et al. [26].

Our findings likely confirm seasonal fluctuation of vitamin D levels [10], but the relationship between vitamin D and AMH concentration is in opposition to some reports [2,21]. A strong negative trend of AMH, both in serum and in FF, was observed with vitamin D concentrations of up to approximately 30 ng/mL; beyond that concentration, the trend is positive but statistically insignificant. Various reasons might explain our outcomes, but similar trends in the relationship between vitamin D and sex hormones have been reported recently. For example, one study [27] found that progesterone concentration was decreased in response to 1,25(OH)_2_D_3_. Furthermore, the authors hypothesize that excess vitamin D may even cause a reduction in this important hormone and consequently have a severe detrimental role during early pregnancy.

With gestational age, liver synthetic function increases, as does the biologically active 1,25(OH)_2_D_3_ metabolite. However, this rise may result from the availability of 25(OH)D [6]. 

During pregnancy, high estrogen levels are responsible for the liver production of many proteins and among them the vitamin D binding protein (DBP). Higher concentrations of binding proteins diminish the level of free vitamin D. Ovarian hormonal controlled hyperstimulation is also associated with high estrogen levels (the mean estradiol serum level in our study was 1269+/− 754 pg/mL). The question that appears here is whether long-lasting hyperstimulation with elevated estradiol levels may influence the level of vitamin D in dependency of DBP availability. However, the average half-life of 25(OH)D in blood is around 3 weeks [7] and hormonal stimulation in our group lasted no longer than 13 days (data not shown).

Total 25(OH)D levels depend on DBP levels that may vary in different conditions and populations [6]. We did not measure DBP concentration, nor liver and renal function during the study. Also, as shown by Tsuprykov et al. [7], free, but not total 25(OH)D characterizes vitamin D status in pregnancy with higher sensitivity.

This is probably one of the limitations in our study. Vitamin D behaves in a similar way to hormones, so to assess vitamin D status among infertile women, we could measure free instead of total 25(OH)D.

The precise steroidogenic signaling cascade of vitamin D is not still well-described in the literature. As stated by Parikh et al. [28], 1,25(OH)_2_D_3_ stimulated progesterone and estradiol production. Together with insulin, 1,25(OH)_2_D_3_ increased estradiol production by 60%. 1,25(OH)_2_D_3_ used alone also influenced IGFBP-1 production, but together with insulin the activity was changed towards inhibition of IGFBP-1 production.

Although, in studies by Aleyasin et al. [29], serum and follicular levels of vitamin D did not differ with the cause of infertility, some associations were found between serum levels of 25(OH)D and PCOS, where 25(OH)D levels increased to compensate for insulin resistance. In our study, PCOS cases were not enrolled.

Likewise, it is possible that physiological levels of vitamin D have a beneficial role in endometrial receptivity, while an excess of vitamin D plays a detrimental role in ovarian homeostasis, disturbing oocyte development and consequently embryo quality [30]. This may explain why decreased expression of vitamin D binding protein (VDBP) in the FF was associated with improved IVF outcomes [31], and why women with serum 25(OH)D <20 ng/mL had higher fertilization, pregnancy, and miscarriage rates than those with levels ≥20 ng/mL [32]. However, Fabris et al. [9] showed, in studies applicable to oocyte donors, that vitamin D levels were unrelated to ovarian reserve, ovarian response after hormonal stimulation, and egg quality. Ozkan et al. [17] reported that, in vitamin-D-deficient animals, hypocalcemia associated with vitamin D deficiency is responsible for reduced fertility, as it is known that vitamin D increases intracellular Ca^2+^. Probably, vitamin D influence on implantation and successful pregnancy outcome results more from immunomodulation via T lymphocytes and NK (natural killers) cells than from its connections with ovarian steroidogenesis. Immune cells also express the 1-α-hydroxylase enzyme that is necessary to convert vitamin D into the active 1,25(OH)_2_D_3_ form [33]. Moreover, immuno-modulating activity may be more crucial in aspects of endometrial receptivity.

We hope that the number of patients, 53, was enough to detect the trend between the vitamin D and AMH relationship (following the applied calculation, the minimum sample size required for the present study, given the desired significance level and statistical power, was 49 patients). We will verify observed results in a larger group of patients, which will definitely improve the power of a statistical test (further examination of the patients is in progress). Nevertheless, our results and the cited reports support the hypothesis that vitamin D has either a minor, or somewhat negative, potential to modify AMH production [10,26]. This could have important therapeutic implications and will be verified in a larger observation group. It is necessary to highlight that embryo quality, its potential to implant, and subsequent pregnancy are strongly dependent on many factors (e.g., sperm and egg quality, endometrial receptivity). It seems that both AMH and vitamin D influence human fertility, but their relationships are rather multidimensional.

## 5. Conclusions

Our study confirms results on seasonal serum changes in vitamin D, and also shows novel aspects of the relationship between vitamin D and AMH.

During infertility treatment, vitamin D may be capable of reducing AMH production.

Because we observed a negative trend in the relationship between AMH with vitamin D for vitamin D concentrations up to 30 ng/mL, according to our selected statistical approach, we suggest the segmentation of this relationship.

Because our clinical study findings conflict with other results in the related literature, they will be verified in a larger group of patients.

## Figures and Tables

**Table 1:** Seasonal differences in vitamin D (total) and other biomarkers.

		Overall	1st Semester	2nd Semester	*t*-Test
Origin	Biomarker	Mean ± SD	Mean ± SD	Mean ± SD	*p*-value
**Serum**	vitamin D total (ng/mL)	29.7 ± 13.3	26.3 ± 13.2	34.2 ± 12.6	0.0363
	AMH (ng/mL)	3.68 ± 2.58	4.52 ± 2.90	2.67 ± 1.72	0.0165
	FSH (mIU/mL)	6.87 ± 1.74	6.61 ± 1.79	7.25 ± 1.66	0.2476
	LH (mIU/mL)	6.12 ± 2.16	6.25 ± 1.74	5.94 ± 2.66	0.6611
	estradiol (pg/mL)	1269 ± 754	1447 ± 662	1076 ± 814	0.0957
**Follicular Fluid**	AMH (ng/mL)	3.01 ± 1.90	3.49 ± 2.14	2.55 ± 1.62	0.0848
	FSH (mIU/mL)	4.75 ± 2.04	4.34 ± 1.73	4.87 ± 2.03	0.3255
	LH (mIU/mL)	0.81 ± 0.82	0.74 ± 0.66	0.77 ± 0.90	0.9174
	estradiol (µg/mL)	542 ± 419	492 ± 346	568 ± 496	0.5344

AMH- anti-Müllerian hormone, FSH- follicle stimulating hormone, LH- luteinizing hormone, SD- standard deviation.

**Table 2:** Baseline descriptive statistics of the patients.

Vitamin D Total	≤30 ng/mL	>30 ng/mL	*t*-test
Patient Characteristics	Mean ± SD	Mean ± SD	*p*-value
Age	34.1 ± 4.2	35.4 ± 4	0.2782
BMI (kg/m^2^)	22.5 ± 2.9	21.8 ± 2.8	0.5208
AMH (ng/mL) (serum)	4.14 ± 3.05	3.18 ± 1.90	0.2137
AMH (ng/mL) (follicular fluid)	3.63 ± 2.14	2.32 ± 1.32	0.0094

AMH- anti-Müllerian hormone, BMI- body mass index, SD- standard deviation.

**Table 3:** Pearson’s linear correlations for total vitamin D and AMH concentrations.

AMH	*r* (CI 95%)	*p*-value
serum	−0.19 (−0.46, 0.12)	0.2211
follicular fluid	−0.28 (−0.51, 0.02)	0.0391

AMH- anti-Müllerian hormone, CI- confidence interval.

**Table 4:** Segmented regression of AMH concentrations in relation to vitamin D concentration. (AMH-anti-Müllerian hormone, CI- confidence interval)

AMH Origin	Regression Parameter	Mean (CI 95%)	*p*-value
**Serum**	change-point	31 (15,47)	0.0001
	slope I	−0.14 (0.00, 0.28)	0.0605
	slope II	0.05 (−0.11, 0.21)	0.5017
**Follicular Fluid**	change-point	33 (15, 51)	0.0003
	slope I	−0.09 (−0.17, −0.01)	0.0340
	slope II	0.02 (−0.10, 0.14)	0.7432
